# Nutrition transition and double burden of malnutrition in Africa: A case study of four selected countries with different social economic development

**DOI:** 10.3934/publichealth.2020035

**Published:** 2020-06-30

**Authors:** Teresia Mbogori, Kilee Kimmel, Mengxi Zhang, Jay Kandiah, Youfa Wang

**Affiliations:** 1 Department of Nutrition and Health Sciences, Ball State University, 2000 W University Avenue, Muncie, Indiana 47306, USA; 2 Systems-Oriented Global Childhood Obesity Intervention Program, Fisher Institute of Health and Well-being, Ball State University, 2000 W University Avenue, Muncie Indiana 47306, USA

**Keywords:** Africa, malnutrition, obesity, overweight, social economic status

## Abstract

**Background:**

Disease and lifestyle patterns have been changing rapidly especially in Africa due to transformation in economic development and urbanization. Research on the magnitude and consequences of these transformations in Africa is limited. This study investigates the shifts in nutritional status in children and adults in four selected low-, middle- and high-income countries in Africa, identifies factors associated with the shifts, and provides recommendations for future studies.

**Methods:**

Malawi, Kenya, Ghana, and South Africa were selected based on their Gross Domestic Product (GDP). Nationally representative data were obtained from the 2017 Global Nutrition Report, Demographic Health Surveys (DHSs), and the World Health Organization (WHO) database. Prevalence of underweight, overweight, and obesity were assessed and compared across the countries, gender, residence, and over time. Results: South Africa had the highest GDP and largest prevalence of overweight and obesity rates in children < 5 years old and adults > 18 (13.3% and 51.9%, respectively). Malawi, with the lowest GDP, had the highest stunting rate (37.0%). In all 4 countries, but most notably in South Africa, trends indicated that the increasing prevalence of overweight and obesity was much greater than the declining rate of underweight. Malawi, Kenya, and Ghana had a slight decline in overweight prevalence in children under 5 years.

**Conclusions:**

Nutritional shifts are occurring in Africa and seem to be heavily influenced by economic development. The double-burden of malnutrition presents prioritization challenges for policymakers. Attention needs to be shifted towards prevention of obesity, at least in the higher income countries in Africa.

## 1. Introduction

It is important to discern and address the shifts in disease burden and the burden of malnutrition in developing countries, many of which face a “double burden” of undernutrition and increasing obesity prevalence [1]–[3]. Recent research indicates that the prevalence of overweight and obesity in Africa continues to increase despite the high prevalence of hunger and malnutrition [4]. The consequences of these shifts in nutritional status and the double burden of malnutrition are severe and have been studied in low-income countries [5]–[8]. Some of the main consequences include the increased risk of developing chronic diseases such as diabetes, high cholesterol and cardiovascular diseases in adulthood [9],[10], which pose a great impact on health care systems, especially in low-income countries [11]. Unfortunately, in many African countries, the consequences of the shifts in nutritional status have not been extensively documented.

Although nutrition transition is observed globally, the shifts in dietary patterns and physical activity in some low-income countries have been shown to occur more rapidly than in high-income countries [12]. Factors such as improved household economic status, mass media, and urbanization have been shown to be driving forces behind these rapid changes in dietary behaviors [13]. To our knowledge, there are no studies that have compared the shifts in dietary patterns in African countries despite the great differences in social economic status among these nations. Most comparative studies have been mainly conducted in America, Europe and Asia [14]. Therefore, it is imperative that researchers study the extent and consequences of the shifts in nutritional status in African countries and provide policy makers and researchers with policy and programmatic recommendations for dealing with these alterations.

This study aims to: (1) investigate the shifts in nutritional status in children and adults in low-, middle- and high-income countries in Africa; and (2) identify factors associated with the shifts in these countries and provide recommendations for future research and interventions. We selected four countries in Africa to investigate, namely Malawi, Kenya, Ghana and South Africa. These countries were chosen based on their economic development status as determined by using per capita Gross Domestic Product (GDP), level of urbanization, general health, as well as their population size (Table 1). South Africa was considered as high-income; Ghana, higher-middle income; Kenya, lower-middle income; and Malawi, low-income.

## 2. Methods and materials

### 2.1. Data sources

Data were extracted from the 2017 Global Nutrition Report [15] Demographic Health Surveys (DHSs) for Malawi, Kenya, Ghana and South Africa [16]–[19], and the World Health Organization (WHO) Global Health Observatory [20],[21]. The inclusion criteria were: (a) the data were collected from nationally representative surveys; (b) anthropometric measures such as stunting, wasting, underweight, and body mass index (BMI) were used to classify prevalence of underweight, overweight, and obesity; (c) stunting, wasting, underweight, overweight, and obesity were defined using WHO standard measures; and (d) data were collected from 1998 to 2016.

### 2.2. Measures

#### 2.2.1. Demographic and Social Economic Status (SES) characteristics

Data derived from the Global Nutrition Report 2017 included the characteristics of the countries studied, including GDP per capita, population size, ratio of urban to rural population, under-5 mortality rates, and the prevalence of undernourishment.

#### 2.2.2. Measurement and classification of nutritional status

Data on the nutritional status of children < 5 years were derived from the DHSs for each country and included measurements of stunting (height-for-age, Z score < −2SD), wasting (weight-for-height, Z score < −2SD), underweight (weight-for-age, Z score < −2SD), and overweight (weight-for-age Z-score > +2SD). For the adult nutritional status, data were derived from the WHO database and included BMI cut points for underweight, overweight and obesity, and obesity (< 18.0 kg/m^2^, ≥ 25.0 kg/m^2^, and ≥ 30.0 kg/m^2^, respectively).

### 2.3. Statistical analysis

To examine the shifts from under- to over-nutrition and to facilitate comparisons across the countries, we calculated a set of ratios using the combined overweight and obesity prevalence against the prevalence of underweight in each country. We also calculated the ratios by rural/urban residence and by gender.

## 3. Results

The demographic, economic, and social development characteristics are presented in Table 1. Malawi had the lowest GDP per capita, total population, and percent of urbanized population, while having the highest prevalence of undernourished individuals (26.3%). Meanwhile, South Africa had the highest GDP per capita, total population, the greatest urbanization, and the lowest overall prevalence of undernourished individuals (6.1%).

### 3.1. Current prevalence of malnutrition in children and adults

#### 3.1.1. Children under 5 years

As shown on Table 2, Malawi had the highest levels of stunting (37%) and underweight (11.7%) and Ghana had the highest prevalence of wasting (4.7%) in 2014. South Africa had the highest prevalence of overweight and obesity (13.3%) while Ghana had the lowest (2.6%). Although South Africa had the highest overweight and obesity rate, the prevalence rate of stunting was also considerably higher at 27.4%, than in Kenya and Ghana. South Africa had the highest overweight and obesity to underweight ratio at 2.25 whereas the Ghana had the lowest ratio at 0.24. Rural-urban differences were observed in Malawi, Kenya and Ghana where 1.5 times more stunted children lived in the rural as compared to urban areas. More overweight and obese children lived in urban than rural areas in Kenya and Ghana. Interestingly, in Malawi and South Africa, the prevalence of overweight and obesity in rural and urban areas was similar with a ratio of 1.0.

#### 3.1.2. Adults ≥ 18 years

As demonstrated on Table 3, the prevalence of overweight and obesity in adults (≥ 18 years) was higher than that of underweight in all four countries. South Africa had the lowest prevalence of underweight (4.8%) and the highest prevalence of overweight and obesity (51.9%). Kenya had the highest prevalence of underweight (11.9%) while Malawi had the lowest prevalence of overweight and obesity (20.1%). South Africa had the highest overweight and obesity to underweight ratio (10.80), compared to Ghana (3.29), Kenya (1.90), and Malawi (1.78). Men had higher rates of underweight while women had a higher rate of overweight and obesity, in all selected countries.

**Figure 1.**
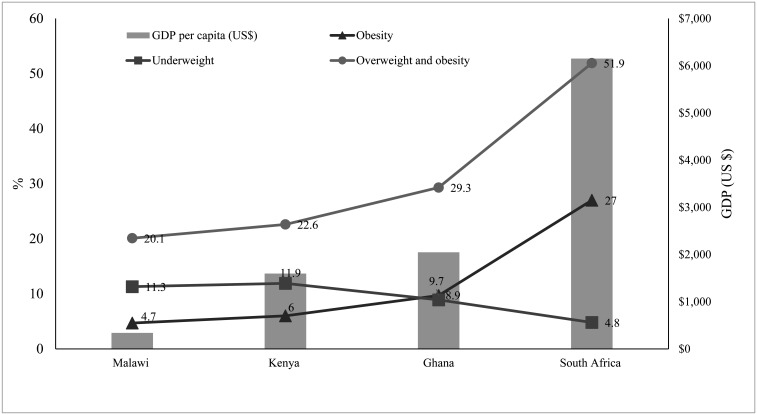
Differences in prevalence (%) of underweight (BMI < 18.5), overweight and obesity (BMI ≥ 25), and obesity (BMI ≥ 30) among adults in 4 selected African countries, by per capita GDP level. Data on the prevalence of underweight, overweight & obesity and Gross Domestic Product per capita were collected from the Global Nutrition Report 2017, and prevalence of obesity were derived from WHO data (2016).

Figure 1 shows the association between the GDP per capita of the selected countries and the nutritional status among adults older than 18 years. Overweight and obesity prevalence increased as the countries' GDP per capita increased whereas the prevalence of underweight decreased as the GDP increased. There was a distinct gap between the prevalence of obesity and underweight. In Malawi, the prevalence of underweight was 2.4 times greater than that of obesity. This gap continued to narrow as GDP increased. In Ghana, the prevalence of obesity and underweight were almost similar with only a 0.8% difference. This gap reversed and widened where the prevalence of obesity in South Africa was 5.6 times higher than the prevalence of underweight.

### 3.2. Shifts in the nutritional status of children and adults from 1998 to 2016

**Figure 2.**
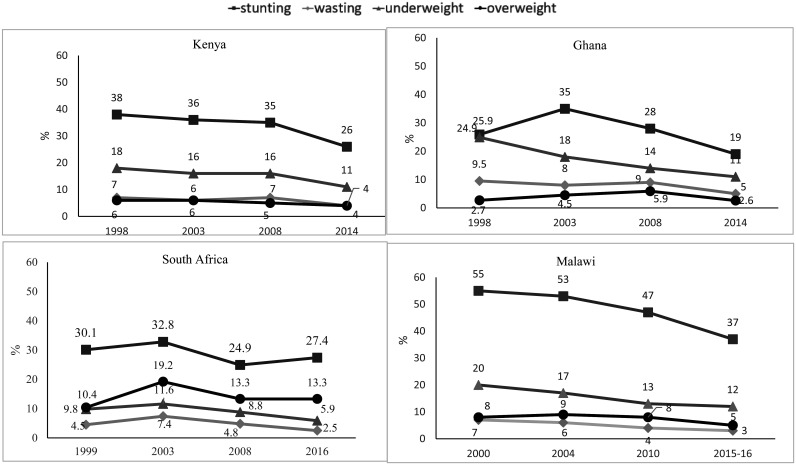
Time trends (1998–2016) in the prevalence (%) of stunting, wasting, underweight, and overweight/obesity among children under 5 years old from 4 selected African countries. Data on prevalence of stunting, wasting, and underweight for Malawi, Kenya, Ghana, South Africa were derived from the Demographic Health Surveys (1998–2016); Data for overweight/obesity in all countries was from WHO database (1998–2016). DHS for Malawi was available from 2000–2016 and for South Africa was available from 1999–2016.

Figure 2 shows the trends of the prevalence of malnutrition among children under 5 years of age in the four countries. Over the 18-year time period, across all four countries, there was a consistent decline in stunting rate. The greatest decline was observed in Malawi (18 percentage points). The trends in the prevalence of wasting, underweight, and overweight remained low, but showed a slight decline in only Malawi, Kenya and Ghana.

**Figure 3.**
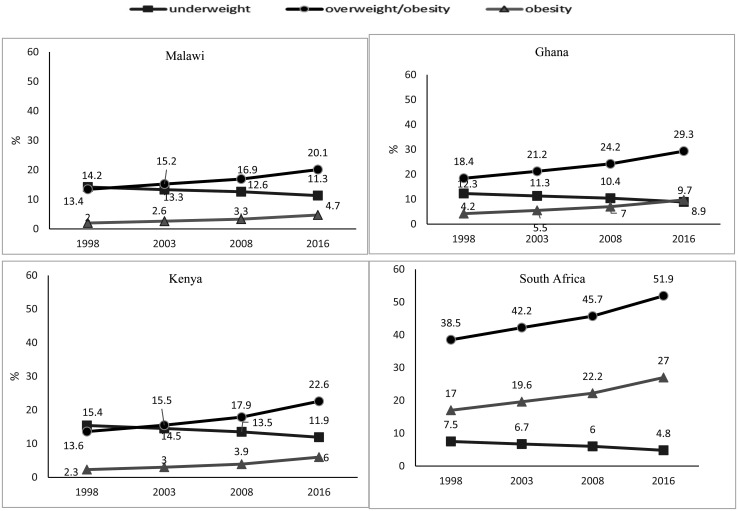
Time trend (1998–2016) in the prevalence (%) of underweight (BMI < 18.5), overweight & obesity (BMI ≥ 25) and obesity (BMI ≥ 30) among adults in 4 selected African countries. The data were derived from the WHO and Global Health Repository dataset (1998–2016).

Figure 3 displays the trends of the prevalence of underweight, overweight, and obesity in adults from 1998–2016. Both overweight and obesity increased gradually from 2000 to 2016 in all four countries. The greatest increase in overweight and obesity were observed in South Africa (11.8%) and the least were observed in Malawi (6.0%). In Ghana and Kenya, the prevalence of overweight and obesity increased by 9.8% and 8.2%, respectively. Similarly, underweight in adults decreased over the years with the highest decline being observed in Kenya (3.1%) and Ghana (3.0%) and the lowest in Malawi (2.5%) and South Africa (2.4%). Overall the magnitude at which the prevalence of overweight and obesity increased over the years was much greater when compared to the rate at which the prevalence of underweight decreased.

**Table 1.** Demographic, Economic, and Social Characteristics of 4 selected African countries: Malawi, Kenya, Ghana, and South Africa.

Country	Income level	GDP per capita^1^ US $, 2017	Population, 2017	Under-5 population 2017	% of population > 65 years, 2017	Under 5 mortality rate per 1,000 live births, 2015	Urban/rural population ratio, 2017	% of population undernourished, 2015–2017
Malawi	Low	338	18 622 000	2 966 000	3	64	0.2	26.3
Kenya	Lower-Middle	1595	49 699 000	7 099 000	3	49	0.4	24.2
Ghana	Lower-Middle	2046	28 833 000	4 124 000	3	62	1.2	6.1
South Africa	Higher-Middle	6151	56 717 000	5 712 000	5	41	1.9	6.1

Data source: Global Nutrition Report 2017 [15]. These countries were selected to represent low-, lower middle-, and high middle-income countries in Africa.

^1^GDP per capita = Gross domestic product (GDP) per capita.

**Table 2.** Prevalence (%) of under- and over-nutrition among children under 5 years old in 4 selected African countries, and the urban/rural differences^1^.

Country		Stunting %	Wasting %	Underweight %	Overweight & Obesity %	Overweight & obesity to underweight ratio
Malawi	All	37.1	2.7	11.7	4.5	***0.38***
	Rural	39.0	2.6	12.3	4.5	***0.37***
	Urban	25.0	3.3	7.9	4.6	***0.58***
	Rural/urban ratio	1.6	0.8	1.6	1.0	
Kenya	All	26.0	4.0	11.0	4.1	***0.37***
	Rural	29.0	4.4	13.0	3.4	***0.26***
	Urban	20.0	3.4	7.0	5.5	***0.79***
	Rural/urban ratio	*1.5*	*1.3*	*1.9*	*0.6*	
Ghana	All	18.8	4.7	11.0	2.6	***0.24***
	Rural	22.0	6.0	13.0	1.9	***0.15***
	Urban	15.0	4.0	9.0	3.4	***0.37***
	Rural/urban ratio	*1.5*	*1.5*	*1.4*	*0.5*	
South Africa	All	27.4	2.5	5.9	13.3	***2.25***
	Rural	29.2	2.5	6.0	13.2	***2.20***
	Urban	25.7	2.4	5.8	13.4	***2.31***
	Rural/urban ratio	*1.1*	*1.0*	*1.0*	*1.0*	

Data source: Prevalence of stunting, wasting, and overweight/obesity was derived from the most recent DHS reports for Malawi; 2015–2016 [18], Kenya; 2014 [17], Ghana; 2014[16], and South Africa; 2016 [19].

^1^Based on best available national recent data during 2014–2016.

^2^Stunting: % height-for-age < −2 SD; Wasting: % weight-for-height < −2 SD; Underweight: % weight-for-age < −2 SD; Overweight/obesity: % weight-for-height > +2 SD

^3^Overweight/obesity to underweight ration was calculated by dividing the prevalence of overweight/obesity in all the children by the prevalence of underweight.

**Table 3.** Prevalence (%) of under- and over-nutrition among adults (> 18 years) and the gender differences in 4 selected African countries^1^.

Country	Gender	Underweight (%, BMI < 18.0)	Overweight & obesity (%, BMI ≥ 25.0).	Obesity (% BMI ≥ 30.0)	Overweight & obesity to underweight ratio	Overweight to obesity ratio
Malawi	All	11.3	20.1	4.7	*1.78*	*4.28*
	Male	13.2	12.8	1.9	*0.97*	*6.74*
	Female	9.4	27.3	7.5	*2.90*	*3.64*
	*Male/ female ratio*	*1.4*	*0.5*	*0.3*		
Kenya	All	11.9	22.6	6.0	*1.90*	*3.77*
	Male	13.9	14.5	2.5	*1.04*	*5.80*
	Female	9.9	30.5	9.4	*3.08*	*3.24*
	*Male/ female ratio*	*1.4*	*0.5*	*0.3*		
Ghana	All	8.9	29.3	9.7	*3.29*	*3.02*
	Male	10.4	20.3	4.1	*1.95*	*4.95*
	Female	7.4	37.9	15.0	*5.12*	*2.53*
	*Male/ female ratio*	*1.4*	*0.5*	*0.3*		
South Africa	All	4.8	51.9	27.0	*10.80*	*1.92*
	Male	6.6	38.6	14.5	*5.85*	*2.66*
	Female	3.2	64.0	38.5	*20.00*	*1.66*
	*Male/ female ratio*	*2.1*	*0.6*	*0.4*		

^1^Based on best available national recent data during 2014–2016.

Data source: prevalence values for underweight, overweight and obesity were derived from WHO 2016 [20].

Male/female ratios were calculated by dividing the male values by the female values.

## 4. Discussion

This study examined the over-time changes and between-group differences in nutritional status during 1998–2016 among four purposively selected countries in Africa with differing social economic statuses (SES). All four nations experienced varied nutrition transition; countries with higher economic status had a greater and more accelerated increase in the prevalence of overweight and obesity. However, slight declines in overweight and obesity were observed in children under 5 years which might be attributed to reduced stunting rates [22],[23]. Rural-urban differences were also found especially in children < 5 years where stunting was more prevalent in rural than urban areas. Additionally, gender differences existed in adults, with females having higher rates of overweight and obesity and males being more undernourished.

The presence of nutrition shifts from under- to over-nutrition was evident and appeared to be more severe in South Africa, a “high-income country” in Africa. Among the 4 countries investigated, South Africa had the highest GDP per capita and 66.0% of the population resided in urban areas. Malawi on the other hand, had the lowest GDP per capita, almost 10 times lower than that of South Africa, and only 17.0% of the population lived in urban areas. Consequently, the prevalence of overweight and obesity in South Africa was 2.5 times that of Malawi. This observation provides additional evidence that economic development and urbanization are key drivers of the worldwide nutrition transition [24]. Living in urban areas is linked to having higher and more diverse sources of income as compared to residing in rural areas [25],[26]. Similar findings on the correlation between urbanization and increased prevalence of overweight and obesity have been noted by previous researchers [27].

Rural-urban differences in nutritional status were observed among children < 5 years in Malawi, Kenya and Ghana. More undernourished children lived in rural than in urban areas, while more overweight and obese children lived in urban than in rural areas. Interestingly, there were no or few rural-urban differences in nutritional outcomes of children < 5 years in South Africa. Despite the extensive evidence of increased prevalence of overweight and obesity in urban areas when compared to rural areas [8],[25],[28], some studies have found that these differences can be attenuated by household SES. A study investigating the rural-urban differences in BMI in 38 low- and middle-income countries found that the mean BMI was higher in urban areas, an association that was attenuated after household SES was accounted for [29]. Although South Africa is among the wealthiest countries in Africa, the distribution of income is uneven. A large percentage of the population do not have access to land and that may explain the high level of urban dwellers [6]. This then could imply that even though there is a large urban population in South Africa, most urban dwellers could be of a lower SES, thereby contributing to the lack of difference in overweight and obesity rates between rural and urban population.

Large gender differences in nutritional status were observed in adults; women from all four countries had a higher prevalence of overweight and obesity whereas men had a higher prevalence of underweight. These findings are in agreement with those from another study showing that in 105 countries, overall, the prevalence of overweight and obesity was higher in women than men [30]. On the contrary, when countries were grouped according to their SES, more men were obese in high-income countries whereas more women were obese in low-income countries. Researchers have attempted to explain the gender differences found in overweight and obesity in women, especially in Africa [31]. Most of these studies point towards culture whereby a larger body size in women is considered more desirable in some African communities than a normal body size [32],[33]. Indeed, one study in Morocco showed intentional strategies for weight gain in women included consumption of appetite enhancers and excessive food, and reduction in physical activity [34].

It is evident from this research that since the late 1990s, all four countries have experienced a steady increase in overweight and obesity rates. Therefore, timely and appropriate policies and programs are needed to combat this increase in overweight and obesity as they continue to reduce the prevalence of under-nutrition. Some international organizations, such as the WHO, and some researchers have provided various recommendations including developing new national policies that regulate food distribution and marketing, especially those targeting children [35],[36]. This study showed the rate at which underweight among adults decreased during 2000-2016 was much slower than the increase rate of overweight and obesity. This was even though African countries, especially the low-income countries have continued to focus on strategies to reduce undernutrition. Unfortunately, such strategies have resulted in increased overweight and obesity in some countries [37]. Therefore, it is critical for policy makers to engage in dialogue with community partners and health professionals to identify solutions that would reduce the prevalence of hunger, while preventing over-nutrition.

The United Nations Sustainable Development Goals (SDGs) have called on countries to develop policies that deal with issues of poverty, hunger, and health [38]. The first three SDGs are: (1) no poverty, (2) zero hunger, and (3) good health and wellbeing [38]. Although these goals are related to managing malnutrition, they are more focused on undernutrition and infectious diseases. Consequently, they may not be adequate for overweight and obesity and non-communicable diseases. Unfortunately, many health-related policies in Africa continue to emphasize on reducing undernutrition and infectious diseases. There are a few established policies that have focused on overweight and obesity. For example, in South Africa, the National Development Plan for 2030 includes a strategy to promote healthy eating and physical activity in schools and within the communities, [39] and the Ghana National Nutrition Policy 2013–2017 emphasizes the need for a healthier diet and physical activity [40]. The Kenya National Control of Non-Communicable Diseases Policy proposes a strategy to improve consumption of healthier diets in an attempt to reduce non-communicable diseases [41]. These policies have yet to be evaluated on their effectiveness in slowing the increased prevalence of overweight and obesity in Africa.

Findings of this study should be viewed considering potential limitations. First, the available data sets confined the availability of variables for analysis. Second, some of the data points were not from the same years and thus limited our comparisons. Third, the available data did not allow us to look at the relationships between urbanization and household SES, and how these have contributed to the shifts in nutritional status. Nevertheless, the main strength of this study is that, to our knowledge, it is the only comprehensive study that compares countries in Africa based on their economic status. Furthermore, we used recent national data which allowed us to present the most current comparisons between the countries.

In conclusion, shifts in nutritional status are present in Africa with an increased prevalence in overweight and obesity in children and adults. These changes are associated with economic development and urbanization, increased intake of a more Westernized diet, and reduced physical activity. Countries with higher SES are more likely to be more urbanized and have higher levels of obesity than countries that have lower SES. In many African countries, current policies focus on reducing hunger. Although there are a few strategies to prevent overweight and obesity in Africa, especially among the lower-income countries, the efficacy of these have yet to be assessed. The repercussions of undernutrition are dire and can lead to lifelong health problems; however, the dilemma of overweight and obesity and the compounding chronic diseases are more detrimental toward the onset of morbidity and mortality. As policy makers strive to achieve the United Nations SDGs related to poverty, hunger and health, ever changing nutritional issues, including the rapidly growing obesity epidemic should also be at center stage.
